# Endobronchial valves for patients with heterogeneous emphysema and without interlobar collateral ventilation: open label treatment following the BeLieVeR-HIFi study

**DOI:** 10.1136/thoraxjnl-2016-208865

**Published:** 2016-12-20

**Authors:** Zaid Zoumot, Claire Davey, Simon Jordan, William H McNulty, Denis H Carr, Matthew D Hind, Michael I Polkey, Pallav L Shah, Nicholas S Hopkinson

**Affiliations:** 1 NIHR Respiratory Disease, Biomedical Research Unit, The Royal Brompton and Harefield NHS Foundation Trust and Imperial College London, London, UK; 2 Respiratory and Critical Care Institute, Cleveland Clinic, Abu Dhabi, UAE

**Keywords:** Emphysema, Lung Volume Reduction Surgery, Bronchoscopy

## Abstract

Outcomes in early trials of bronchoscopic lung volume reduction using endobronchial valves for the treatment of patients with advanced emphysema were inconsistent. However improvements in patient selection with focus on excluding those with interlobar collateral ventilation and homogeneous emphysema resulted in significant benefits in the BeLieVeR-HIFi study compared with sham treated controls. In this manuscript we present data from the control patients in the BeLieVeR-HIFi study who went on to have open label endobronchial valve treatment after completion of the clinical trial (n=12), combined with data from those in the treatment arm who did not have collateral ventilation (n=19). Three months after treatment FEV_1_ increased by 27.3 (36.4)%, residual volume reduced by 0.49 (0.76) L, the 6 min walk distance increased by 32.6 (68.7) m and the St George Respiratory Questionnaire for COPD score improved by 8.2 (20.2) points. These data extend the evidence for endobronchial valve placement in appropriately selected patients with COPD.

**Trial registration number:**

ISRCTN04761234; Results.

## Introduction

Patients with emphysema are breathless because of gas trapping and hyperinflation due to the loss of lung elastic tissue and resultant expiratory airways collapse. Surgical lung volume reduction (LVRS) can improve survival, lung function and quality of life in selected patients with exercise limitation and heterogeneous emphysema.^1^ ^2^ The placement of endobronchial valves (BLVR) as a means to reduce lung volume is a potential alternative to LVRS. BLVR has been shown to improve lung function, reduce chest wall asynchrony and reduce the work of breathing.^3^ ^4^ Atelectasis following BLVR is associated with improved survival.^5^ ^6^ The BeLieVeR-HIFi study, a double-blind sham-controlled trial,^7^ ^8^ found that BLVR led to significant improvements in lung function, exercise capacity and health status at 3 months when performed in patients with a higher chance of developing atelectasis—those with intact interlobar fissures and heterogeneously distributed emphysema. In this research letter we present data from the control patients in the BeLieVeR-HIFi study who went on to have open label endobronchial valve treatment after completion of the clinical trial. We also combine these data with patients from the original treatment arm who had been found to be collateral ventilation negative (CV−) using the Chartis catheter system and completed trial follow-up.

## Methods

The study protocol, design, randomisation, assessments, procedure and details of the participants have been previously published^8^ and further details of the methods and statistical analyses are in online supplementary panel S1.

10.1136/thoraxjnl-2016-208865.supp1 supplementary data

## Results

**Figure 1:**
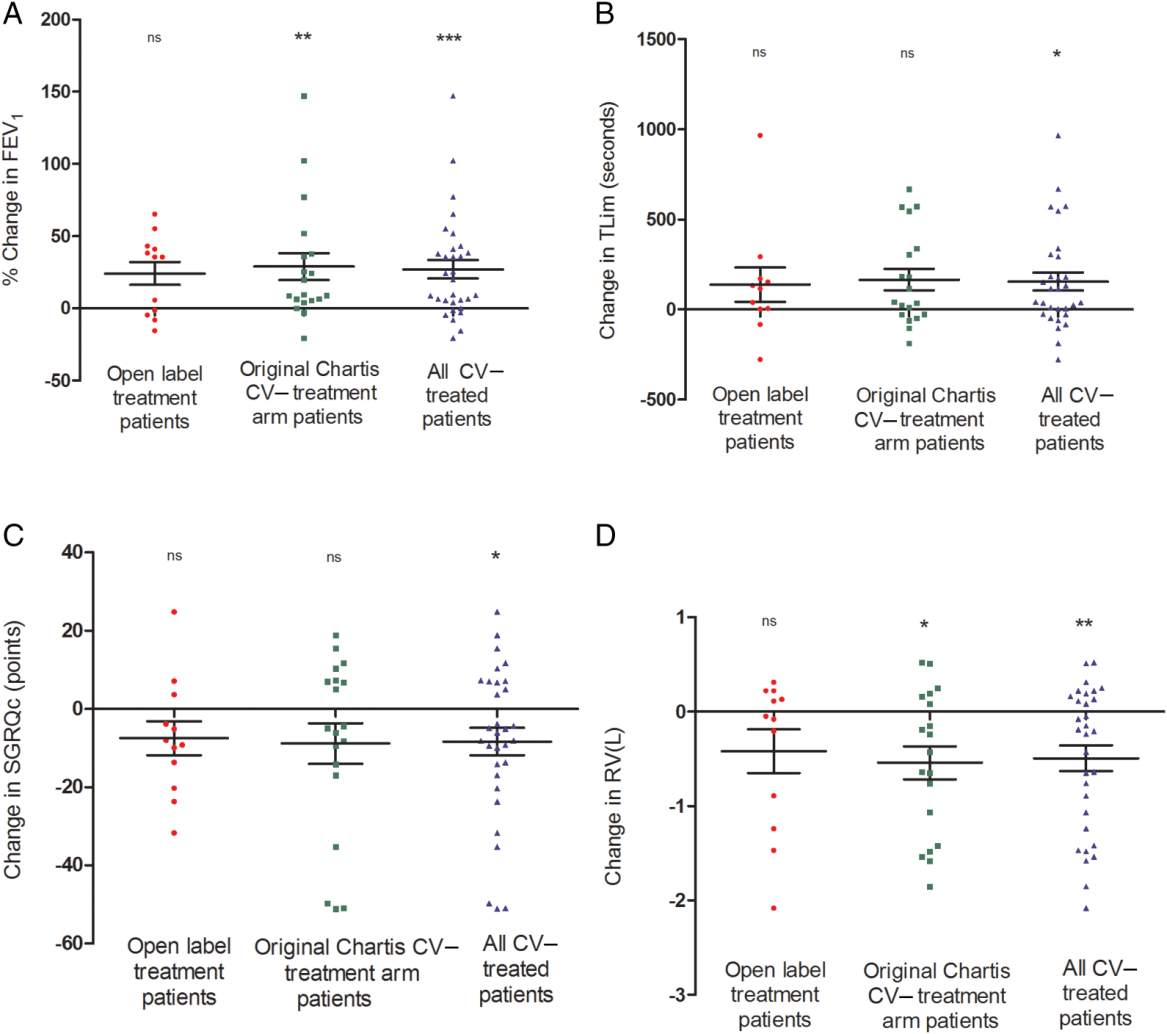
Response to bronchoscopic lung volume reduction in open label treated patients, in the original BeLieVeR-HIFi treated patients who were collateral ventilation negative (CV−) and in both groups combined. (A) FEV_1_; (B) endurance time on cycle ergometry at 70% maximal work rate (Tlim); (C) St George's Respiratory Questionnaire for COPD (SGRQc); (D) Residual volume (RV) assessed by body plethysmography. The p values are for the Wilcoxon signed-rank test. *p<0.05, **p<0.01, ***p<0.001.

Baseline characteristics of the open label treated patients (n=14) are detailed in online supplementary table S1. Three-month follow-up data were available for 12 open label patients. One died 4 days following treatment due to a pneumothorax occurring at their home; one developed a persistent intractable cough necessitating valve removal and did not return for follow-up evaluation. Clinical outcomes are detailed in table 1 and online supplementary table S2, and illustrated in figure 1A–D and online supplementary figure S1A–D. FEV_1_ increased by 24.2 (27.3)% from baseline following endobronchial valve treatment. The patients also experienced statistically significant improvements in carbon monoxide transfer factor and COPD assessment test score as well as measures of exercise capacity. Table 1 also includes data from the 19 CV− patients from the original treatment arm of the BeLieVeR-HIFi trial for whom follow-up data were available (‘original CV− treatment arm patients’) and for the two groups combined (‘all CV− treated patients’) (n=31).

**Table 1:** Change in lung function, health status and exercise tolerance at 90 days

	Open label valve treated patients (n=12)	p Value	Original Chartis CV− treatment arm patients (n=19)	p Value	All CV− treated patients (per Chartis) (n=31)	p Value
%ΔFEV_1_	24.2 (27.3)	0.06	28.9 (40.1)	0.001	27.3 (36.4)	0.0002
ΔFEV_1_ (l)	0.14 (0.20)	0.06	0.23 (0.28)	0.001	0.19 (0.25)	0.0002
%ΔFVC	5.1 (13.0)	0.5	7.51 (16.9)	0.03	6.5 (15.6)	0.02
ΔTLC (l)	−0.23 (0.49)	0.13	−0.37 (0.56)	0.01	−0.33 (0.53)	0.002
ΔRV (l)	−0.42 (0.80)	0.41	−0.54 (0.76)	0.01	−0.49 (0.76)	0.007
ΔRV/TLC %	−3.50 (6.77)	0.10	−4.6 (6.9)	0.03	−4.3 (6.85)	0.004
ΔFRC (l)	−0.28 (0.83)	0.27	−0.42 (0.69)	0.04	−0.38 (0.75)	0.009
ΔTLco (absolute percentage points)	3.5 (6.77)	0.005	3.45 (6.2)	0.02	3.62 (5.16)	0.0007
ΔKco (mmol/min/kPa/l)	0.10 (0.07)	0.007	0.05 (0.07)	0.009	0.07 (0.07)	<0.0001
ΔCAT	−3.9 (5.5)	0.05	−4.2 (10.1)	0.20	−4.1 (8.5)	0.03
ΔSGRQc total	−7.5 (14.9)	0.08	−7.5 (20.8)	0.3	−8.5 (20.2)	0.05
Δ6MWD	29 (48)	0.16	33.2 (80.2)	0.02	32.6 (68.7)	0.01
ΔTLim	138 (312)	0.08	165 (260)	0.07	155 (275)	0.01

Data are presented as mean (SD). The p values are for the Wilcoxon signed-rank test.

6MWD, 6-min walk distance; CAT, COPD assessment test score; Chartis CV−, no interlobar collateral ventilation on Chartis assessment; CV−, collateral ventilation negative; FRC, functional residual capacity; Kco, carbon monoxide transfer coefficient; RV, residual volume; SGRQc, St George's Respiratory Questionnaire for COPD; TLC, total lung capacity; TLco, carbon monoxide transfer factor; Tlim, endurance time on cycle ergometry at 70% of peak workload.

Responder rates for achievement of minimal clinically important differences were similar in the open label patients to those in the original treatment group of the trial (see online supplementary table S3). Eight of 12 patients treated with valves developed atelectasis or complete lobar collapse on CT, and another two had significant volume loss. Details of adverse events are in online supplementary panel S2.

## Discussion

These data further support the view that treating patients with heterogeneous emphysema and without interlobar CV with endobronchial valves leads to improved lung function, exercise capacity and quality of life. The benefits are more impressive where stricter patient selection criteria are employed, although there is still significant variability in response. The improvement in gas transfer is of particular interest as this is the lung function measure most strongly associated with survival in people with COPD.^9^

In the original BeLieVeR-HIFi trial, eligibility for valves was based on the results of CT fissure analysis. CV was measured directly using the Chartis system, but by design patients in the intervention arm were still treated even if they were CV-positive. In this open label follow-up however, patients had had a previous bronchoscopy which confirmed airway anatomy suitable for adequate valve placement and prior Chartis measurements confirming the absence of CV. The proportion of open label treated patients with radiological evidence of volume loss was 83% (10 of 12), higher than the 65% in the original treatment cohort (15 of 23) with rates of responders achieving minimum clinically important differences (MCIDs) in the various outcomes broadly similar to the original group.

The development of bronchoscopic lung volume reduction techniques has been driven by the desire to offer patients safer and cheaper alternatives to LVRS. As patient selection improves, increasing the likelihood of successful lung volume reduction, there is a significantly higher rate of pneumothorax than the 5% reported in earlier trials. In this series the rate was 10.3%, including one fatal event, though others have reported rates of 20–25%.^10^ Although a marker of procedural effectiveness, with eventual clinical benefit in the majority after treatment of the pneumothorax,^11^ pneumothorax can be fatal in people with advanced lung disease and little respiratory reserve. Thus, the mortality risk of BLVR may not be lower than that of surgical intervention, especially when compared with unilateral LVRS.^1^ Pooled data suggest that 70% of pneumothoraces occur within 72 hours^12^ and therefore it may be prudent to observe patients in hospital for 4 days post treatment. Patients who do suffer pneumothoraces are initially managed conservatively and patiently in hospital but may ultimately require valve removal or surgery.^10^ This also reduces the advantage of BLVR over LVRS in terms of hospital length of stay, though the level of dependency during this observation period is low.

Our series highlight that fissure completeness, assessed visually on CT thorax (even by dedicated thoracic radiologists) is not a perfect surrogate for the absence of interlobar CV. Out of 50 patients enrolled in the original trial and all judged to have intact fissures, 8 patients (16%) had positive CV on Chartis assessment.

In conclusion, bronchoscopic lung volume reduction using endobronchial valves leads to clinically significant improvements in lung function, exercise capacity and quality of life in the majority of patients when appropriately selected. The risk of pneumothorax needs to be considered and a period of close observation is recommended. Longer follow-up to assess durability of clinical benefits and effect on survival is needed as well as direct comparison of endobronchial valve placement against surgical approaches.
