# Supplementary material for: Endobronchial valves for patients with heterogeneous emphysema and without interlobar collateral ventilation: open label treatment following the BeLieVeR-HIFi study
Source: Thorax. 2016 Dec 20;72(3):277–9. doi: 10.1136/thoraxjnl-2016-208865 (PMC5339569; doi:10.1136/thoraxjnl-2016-208865)
Supplement: supplementary data [file thoraxjnl-2016-208865supp.pdf]

Online Supplement:

Endobronchial valves for patients with heterogeneous emphysema and without interlobar collateral ventilation – open label treatment following the BeLieVeR-HIFi study

Zaid Zoumot PhD[1,2], Claire Davey MSc[2], Simon Jordan MD[2], William H. McNulty MBBS[2], Dennis H. Carr MD[2], Matthew D. Hind PhD[2], Michael I. Polkey PhD[2], Pallav L. Shah MD[2], *Nicholas S. Hopkinson PhD[2]

[1]NIHR Respiratory Disease, Biomedical Research Unit at the Royal Brompton and Harefield NHS Foundation Trust and Imperial College London, London, UK

[2]Respiratory and Critical Care Institute, Cleveland Clinic Abu Dhabi, UAE

**Corresponding author*

Address The Royal Brompton Hospital,

Fulham Road, London, SW3 6NP, UK

Email n.hopkinson@ic.ac.uk

Telephone +44 2073497775

Fax +44 2073497778

Panel S1: METHODS

The BeLieVeR-HIFi study was a 1:1 randomised, parallel group, double-blind sham bronchoscopy controlled trial of unilateral, endobronchial valve placement (Zephyr valves, PulmonX, California) in patients with heterogeneous emphysema and intact interlobar fissures. The study was approved by the London – Bentham Research Ethics Committee (REC No. 11/LO/1608), the sponsor was Imperial College, London. There was a trial steering group and an independent data monitoring committee. All patients provided written informed consent and were recruited between March 2012 and September 2013. The procedures were performed using moderate sedation with Midazolam and Alfentanyl in the bronchoscopy suite at the Royal Brompton Hospital. Endobronchial valves were placed to occlude all segmental bronchi leading to the target lobe

Patients with optimally medically managed and stable severe or very severe COPD were included (forced expiratory volume in one second (FEV_1) <50% predicted, residual volume (RV) >150% predicted, 6 minute walk distance (6MWD) <450m, and significant breathlessness (MRC dyspnoea score $\geq$3)). Their CT scans demonstrated heterogeneous emphysema with a defined target lobe, and intact adjacent interlobar fissures. Assessments were performed at baseline and 3 months after bronchoscopy, by an assessment team blind to treatment allocation. Assessments included complete lung function testing, endurance cycle ergometry with metabolic measurements at 70% of their maximal workload determined on an initial incremental test, a 6MWD test, and quality of life questionnaires (SGRQc[1] and CAT[2 3]).

Target lobe volume change was assessed by a thoracic radiologist (DC) and scored as follows:

0 – No change,

1 - Some volume loss (Fissures shift)

2- Segmental atelectasis (Band of collapsed lung)

3 – Complete atelectasis (Complete collapse).

Open Label group

Measurements of collateral ventilation using the Chartis^{TM} (PulmonX, California) balloon catheter system were made in all participants during the initial sham bronchoscopy. At the 3 months end of study visit, all treatment options available to the control group patients were discussed. Only patients where collateral ventilation was not detected (CV –ve) were offered open label valve placement. Treatment was performed within 2 weeks of the end of study visit. Of 25 control patients, 14 had BLVR treatment and of the remaining 11, two had died, four had positive collateral ventilation during Chartis assessment (of these two had no further treatment, one had LVRS and one was enrolled into another bronchoscopic LVR trial), and of the five without collateral ventilation on Chartis assessment two preferred LVRS, one had recurrent infections and much airway secretions on initial bronchoscopy and thus valves were avoided in favour of LVRS, one was felt to have a very high risk of pneumothorax due to the presence of ipsilateral paraseptal bullae in the non-target lobe, and one was found to have airway anatomy which did not support endobronchial valve placement.

Statistical Analysis

The primary endpoint in this follow-up study is the difference in the percentage change in FEV_1 measured 3 months post procedure. Secondary endpoints included: (1) change in the

Residual Volume (RV), (2) change in endurance time (Tlim) on cycle ergometry at 70% of maximum achieved workload, (3) change in six minute walk distance, and (4) changes in health status (CAT, SGRQc).

Statistical analysis performed by ZZ using PASW (Predictive Analytics Software; IBM) version 20 for Windows. Categorical data are presented as percentages and comparisons done using the Pearson chi-squared test. Normally distributed numeric data are presented as mean (SD). Non-normally distributed numeric data are presented as median (IQR). Statistical analysis for differences between baseline values (at the final control visit) and 3 months following endobronchial valve treatment was assessed by Wilcoxon signed rank tests. A p value <0·05 was taken to indicate statistical significance. For responder analyses minimum clinically important differences (MCID) were pre-specified as a 15% increase for FEV_1, 350mls reduction for the residual volume[4], 4 points decrease for the SGRQc[1 5], 2 points decrease for the CAT[3 6] an increase of 105 seconds for endurance cycle Tlim[7] and an increase of 26m for the 6MWD[8].

Table S1 Baseline characteristics of study participants

	Open label valve treated patients (n=14)	Original treatment Arm (n=25)	All valve treated patients (n=39)
Age	63.9 (7.2)	62.3 (7.0)	62.7 (6.9)
Gender (M/F)	7/7	17/8	24/15
BMI	24.1 (5.0)	24.5 (5.1)	24.4 (4.9)
PYH	55.6 (27.0)	56 (26)	55.5 (25.4)
Exacerbation rate/yr	2.79 (2.36)	3 (3)	3.24 (2.74)
FEV_1 (L)	0.83 (0.34)	0.93 (0.35)	0.89 (0.33)
FEV_1%pred	31.1 (9.7)	31.6 (10.2)	32.0 (10.3)
TLC %pred	141.0 (15.5)	132 (12)	135.1 (13.6)
RV %pred	241 (47)	219 (39)	226.5 (41.8)
RV%TLC	64.1 (8.1)	60.23 (8.06)	61.7 (8.0)
TL_{CO} %pred	33.5 (11.5)	33.8 (10.8)	34.1 (10.9)
Kco %pred	43.7 (13.9)	45.8 (12.8)	45.9 (13.5)
$PaCO_2$ (kPa)	4.99 (0.69)	4.81 (0.86)	4.85 (0.79)
PaO_2 (kPa)	9.32 (0.61)	9.74 (1.45)	9.56 (1.20)
MRC	3.64 (0.50)	4 (1)	3.59 (0.50)
CAT	25.7 (5.2)	24 (5)	24.7 (5.5)
SGRQc - total	69.7 (12.4)	67.8 (13.2)	69.0 (12.8)
6MWD (m)	335.3 (80.4)	342.3 (94.6)	337.2 (89.8)
Tlim (s)	286.4 (200.3)	306.1 (166.7)	299.3 (176.4)

Data are presented as mean (SD). BMI, body mass index; PYH, tobacco pack year history; FEV_1, forced expiratory volume in 1 second; TLC, total lung capacity; RV, residual volume, TLco, carbon monoxide transfer factor; MRC, Medical Research Council dyspnoea score; CAT, COPD assessment test score; SGRQc, St George's Respiratory Questionnaire for COPD; PYH, pack year smoking history.; 6MWD, six minute walk distance; Tlim, endurance time on cycle ergometry at 70% of peak workload.

Table S2 Change in lung function, health status and exercise tolerance at 90 days

	Open label valve treated patients (n=12)	p value	Original Chartis CV –ve treatment arm patients (n=19)	p value	All CV –ve treated patients (per Chartis) (n=31)	p value
%ΔFEV_1	35.6 (-2.2 to 41.6)	0.06	9.4 (6.0 to 36.8)	0.001	19.8 (4.8 to 39.7)	0.0002
ΔFEV_1 (L)	0.21 (-0.18 to 0.29)	0.06	0.13 (0.05 to 0.38)	0.001	0.18 (0.04 to 0.32)	0.0002
%ΔFVC	0.46 (-5.4 to 16.6)	0.5	4.8 (1.4 to 11.3)	0.03	3.1 (-4.0 to 13.8)	0.02
ΔTLC (L)	-0.05 (-0.26 to 0.01)	0.13	-0.32 (-0.65 to 0.05)	0.01	-0.16 (-0.60 to 0.03)	0.002
ΔRV (L)	-0.06 (-0.98 to 0.15)	0.41	-0.43 (-1.25 to 0.12)	0.01	-0.21 (-1.16 to 0.15)	0.007
ΔRV/TLC %	-2.9 (-7.9 to 2.5)	0.10	-4.7 (-8.4 to 0.3)	0.03	-3.4 (-8.4 to 1.2)	0.004
ΔFRC (L)	-0.02 (-0.66 to 0.16)	0.27	-0.24 (-1.25 to 0.04)	0.04	-0.22 (-0.94 to 0.09)	0.009
ΔTLco (absolute percentage points)	3.3 (1.8 to 5.5)	0.005	2.9 (-0.5 to 5.5)	0.02	3.3 (0.9 to 5.6)	0.0007
ΔKco ($mmol.min^{-1}.kPa^{-1}.L^{-1}$)	0.11 (0.07 to 0.15)	0.007	0.06 (0.01 to 0.10)	0.009	0.08 (0.01 to 0.13)	<0.0001
ΔCAT	-2.5 (-8.5 to -0.5)	0.05	-5.0 (-8.5 to 1.0)	0.20	-4.0 (-8.5 to 1.0)	0.03
ΔSGRQc total	-8.6 (-15.3 to -2.0)	0.08	-4.9 (-15.5 to 7.1)	0.3	-6.1 (-15.5 to 6.9)	0.05
Δ6MWD	16.5 (-6.75 to 70.5)	0.16	30.0 (12.0 to 76.5)	0.02	30.0 (-2.5 to 75.0)	0.01
ΔTLim	116.0 (4.0 to 161.5)	0.08	42.0 (-26.5 to 321.0)	0.07	79.0 (-18.8 to 264.5)	0.01

Data are presented as median (IQR). P values are for Wilcoxon signed rank test. FEV_1, forced expiratory volume in 1 second; FVC, forced vital capacity; TLC, total lung capacity; RV, residual volume; TLco, carbon monoxide transfer factor; Kco, carbon monoxide transfer coefficient; MRC, Medical Research Council dyspnoea score; CAT, COPD assessment test score; SGRQc, St George's Respiratory Questionnaire for COPD; PYH, pack year smoking history.; 6MWD, six minute walk distance; Tlim, endurance time on cycle ergometry at 70% of peak workload; Chartis CV –ve: No interlobar collateral ventilation on Chartis assessment.

Table S3 Responder rates according to lung function, health status and exercise criteria

	Cross-over valve treated patients (n=12)	Original treatment arm Chartis CV –ve patients (n=19)	AllChartis CV –ve valve treated patients (n=31)
FEV_1 ≥15% improvement	7 (58%)	9 (47%)	16 (53%)
RV ≥0.35L reduction	4 (33%)	10 (53%)	14 (45%)
6MWD ≥26m improvement	6 (50%)	11 (58%)	17 (55%)
Endurance cycle time ≥105 secs improvement	6 (50%)	9 (47%)	15 (48%)
SGRQc ≥4 points reduction	8 (67%)	11 (58%)	19 (61%)
CAT ≥2 points reduction	6 (50%)	12 (63%)	18 (58%)

FEV_1 forced expiratory volume in one second, RV residual volume, 6MWD six minute walk distance, SGRQc St George's respiratory questionnaire, CAT COPD assessment test score, Chartis CV –ve: No interlobar collateral ventilation on Chartis assessment.

Panel S2: Adverse events

There was one death secondary to a pneumothorax directly attributable to treatment with endobronchial valves. Changes to our admission and follow-up protocols were subsequently implemented as described above. There was one other pneumothorax in an open label patient. This occurred 5 days post treatment (one day after discharge), and the patient was treated conservatively with intercostal chest tube drainage. He had excellent clinical outcomes at his 3 months follow-up visit. One patient developed a persistent cough necessitating bronchoscopy and valve removal. Two other patients required repeat bronchoscopy to investigate clinical deteriorations, and in one case valves were repositioned and the other mucus plugs cleared with good outcomes in both cases.

Figure S1 (A-D): Response to valve placement CV –ve patients.

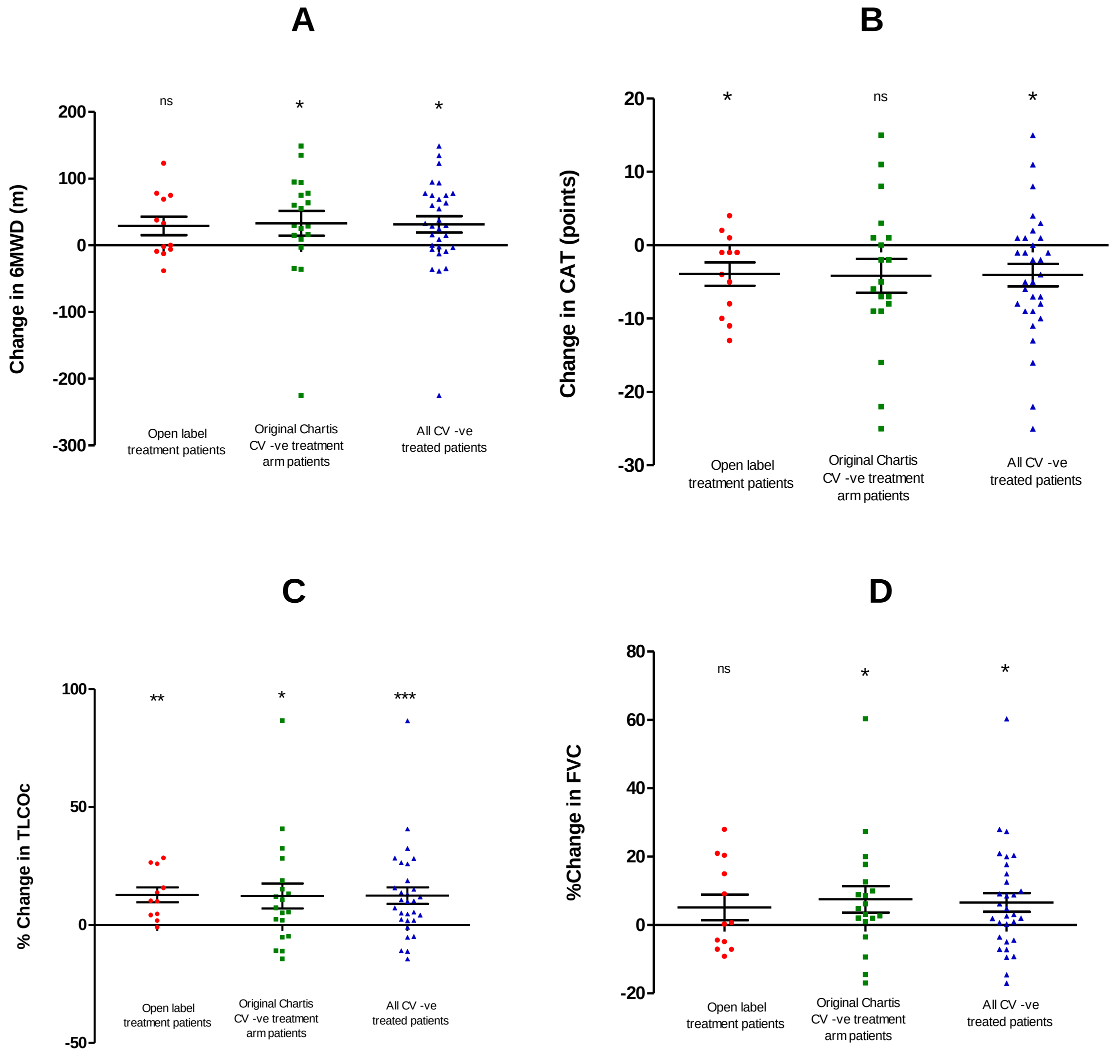

Response to bronchoscopic lung volume reduction in open label treated patients, in the original BeLieVeR-HIFi treated patients who were collateral ventilation negative and in both groups combined. Panel A, six minute walk distance; B COPD assessmsent test score; C TLco carbon monoxide transfer factor; D forced vital capacity. * P<0.05, ** P<0.01, *** P<0.001.

Panel S3: Limitations of the study

Limitations of this extension study include the small sample size and open-label and unblinded nature of the treatment and follow-up. Furthermore, analysis does not account for missing data from patient who died. However the primary and most secondary outcomes were objective rather than subjective. All treatments were undertaken at a single highly specialised centre with extensive experience of treating patients with end stage emphysema using LVRS and a variety of bronchoscopic lung volume reduction techniques, as part of clinical trials and in routine clinical care. The set-up at our centre includes a pre-existing multidisciplinary team that meets on a weekly basis involving dedicated thoracic radiologists, thoracic surgeons, pulmonologists and physiotherapists. It may not be possible to achieve similar outcomes at other centres without this infrastructure or clinical experience

REFERENCES

1. Meguro M, Barley EA, Spencer S, et al. Development and validation of an improved, COPD-specific version of the St George's respiratory questionnaire*. CHEST Journal 2007;**132**(2):456-63 doi: 10.1378/chest.06-0702[published Online First: Epub Date]|.
2. Jones PW, Harding G, Berry P, et al. Development and first validation of the COPD Assessment Test. Eur Respir J 2009;**34**(3):648-54 doi: 10.1183/09031936.00102509[published Online First: Epub Date]|.
3. Dodd JW, Hogg L, Nolan J, et al. The COPD assessment test (CAT): response to pulmonary rehabilitation. A multicentre, prospective study. Thorax 2011;**66**(5):425-29 doi: 10.1136/thx.2010.156372[published Online First: Epub Date]|.
4. Hartman JE, ten Hacken NHT, Klooster K, et al. The minimal important difference for residual volume in patients with severe emphysema. European Respiratory Journal 2012;**40**(5):1137-41 doi: 10.1183/09031936.00219111[published Online First: Epub Date]|.
5. Schünemann HJ, Griffith L, Jaeschke R, et al. Evaluation of the minimal important difference for the feeling thermometer and the St. George's Respiratory Questionnaire in patients with chronic airflow obstruction. Journal of Clinical Epidemiology 2003;**56**(12):1170-76 doi: 10.1016/s0895-4356(03)00115-x[published Online First: Epub Date]|.
6. Kon SSC, Canavan JL, Jones SE, et al. Minimum clinically important difference for the COPD Assessment Test: a prospective analysis. The Lancet Respiratory Medicine 2014;**2**(3):195-203 doi: http://dx.doi.org/10.1016/S2213-2600(14)70001-3[published Online First: Epub Date]|.
7. Casaburi R. Factors Determining Constant Work Rate Exercise Tolerance in COPD and their Role in Dictating the Minimal Clinically Important Difference in Response to Interventions. COPD: Journal of Chronic Obstructive Pulmonary Disease

2005;**2**(1):131-36 doi: doi:10.1081/COPD-200050576[published Online First: Epub Date]|.

8. Puhan MA, Chandra D, Mosenifar Z, et al. The minimal important difference of exercise tests in severe COPD. European Respiratory Journal 2011;**37**(4):784-90 doi: 10.1183/09031936.00063810[published Online First: Epub Date]|.
